# Suitability of administrative claims databases for bariatric surgery research – is the glass half-full or half-empty?

**DOI:** 10.1186/s12874-020-01106-8

**Published:** 2020-09-07

**Authors:** Xiaojuan Li, Kristina H. Lewis, Katherine Callaway, J. Frank Wharam, Sengwee Toh

**Affiliations:** 1 Department of Population Medicine, Harvard Medical School and Harvard Pilgrim Health Care Institute, 401 Park Drive, Suite 401 East, Boston, MA 02215 USA; 2 OptumLabs Visiting Fellow, Eden Prairie, MN USA; 3 Division of Public Health Sciences, Wake Forest University School of Medicine, Winston-Salem, NC USA

**Keywords:** Bariatric surgery, Body mass index, Healthcare administrative claims, Predictive value of tests, Sensitivity and specificity, Validation study

## Abstract

**Background:**

Claims databases are generally considered inadequate for obesity research due to suboptimal capture of body mass index (BMI) measurements. This might not be true for bariatric surgery because of reimbursement requirements and changes in coding systems. We assessed the availability and validity of claims-based weight-related diagnosis codes among bariatric surgery patients.

**Methods:**

We identified three nested retrospective cohorts of adult bariatric surgery patients who underwent adjusted gastric banding, Roux-en-Y gastric bypass, or sleeve gastrectomy between January 1, 2011 and June 30, 2018 using different components of OptumLabs® Data Warehouse, which contains linked de-identified claims and electronic health records (EHRs). We measured the availability of claims-based weight-related diagnosis codes in the 6-month preoperative and 1-year postoperative periods in the main cohort identified in the claims data. We created two claims-based algorithms to classify the presence of severe obesity (a commonly used cohort selection criterion) and categorize BMI (a commonly used baseline confounder or postoperative outcome). We evaluated their performance by estimating sensitivity, specificity, positive predictive value, negative predictive value, and weighted kappa in two sub-cohorts using EHR-based BMI measurements as the reference.

**Results:**

Among the 29,357 eligible patients identified using claims only, 28,828 (98.2%) had preoperative weight-related diagnosis codes, either granular indicating BMI ranges or nonspecific denoting obesity status. Among the 27,407 patients with granular preoperative codes, 12,346 (45.0%) had granular codes and 9355 (34.1%) had nonspecific codes in the 1-year postoperative period. Among the 3045 patients with both preoperative claims-based diagnosis codes and EHR-based BMI measurements, the severe obesity classification algorithm had a sensitivity 100%, specificity 71%, positive predictive value 100%, and negative predictive value 78%. The BMI categorization algorithm had good validity categorizing the last available preoperative or postoperative BMI measurements (weighted kappa [95% confidence interval]: preoperative 0.78, [0.76, 0.79]; postoperative 0.84, [0.80, 0.87]).

**Conclusions:**

Claims-based weight-related diagnosis codes had excellent validity before and after bariatric surgical operation but suboptimal availability after operation. Claims databases can be used for bariatric surgery studies of non-weight-related effectiveness and safety outcomes that are well-captured.

## Background

Bariatric surgery is the most effective treatment for severe obesity, a risk factor for many health conditions including cardiovascular diseases and death [1]. Patients who undergo bariatric surgery can achieve effective weight loss and remission of many comorbidities [2, 3]. However, between 2011 and 2018, only 1% of adults with severe obesity in the United States received bariatric surgery in a given year [4, 5]. With the persistent increase in the prevalence of obesity and considerable shift in the type of bariatric surgical operations performed over the last decade [5], it is important to evaluate the long-term comparative effectiveness and safety of different operations.

Administrative claims databases are an important real-world data source in comparative effectiveness and safety research. These databases often provide large and demographically diverse study populations at a fraction of the cost compared to other data sources [6]. Claims databases also capture most, if not all, medically attended events including hospitalizations and procedures performed. However, claims databases are generally considered inadequate for obesity-related research due to the lack of body mass index (BMI) measurements and the underuse and poor validity of weight-related diagnosis codes [7–10]. This limitation may not necessarily apply to bariatric surgery research because most health insurers in the United States require surgical facilities to receive approval to perform a given bariatric operation (a.k.a., “prior authorization”). This process involves documentation of eligibility, including having a BMI measurement ≥40 kg/m^2^, or a BMI measurement ≥35 kg/m^2^ with at least 1 obesity-related co-morbidity, which are typically converted into diagnosis codes in the patient’s medical record and reimbursement claims [11–13]. In addition, the specific International Classification of Diseases, Ninth Revision, Clinical Modification (ICD-9-CM) weight-related diagnosis codes denoting BMI ranges became available in 2006, with a subset of diagnosis codes indicating BMI ≥40 kg/m^2^ becoming effective in January 2011. The more granular ICD-10-CM codes became effective in October 2015. These coding changes and the prior authorization requirements may considerably improve the availability and validity of weight-related diagnosis codes in claims databases among bariatric surgery patients.

In this study, we evaluated the availability and validity of weight-related diagnosis codes before and after bariatric surgical operations in a large claims database linked to an electronic health record (EHR) database with actual BMI measurements.

## Methods

### Data source

This study used data from the OptumLabs® Data Warehouse (OLDW), which contains linked de-identified administrative claims data for commercially insured and Medicare Advantage enrollees, and de-identified EHR data that has been normalized and standardized into a single database. As of May 2019, the database contains longitudinal health information on over 200 million lives, 137 million in claims, 88 million in the EHR, and 26 million in the linked component since 2007 from a diverse mixture of ages, ethnicities, and geographical regions across the United States [14]. The claims data component includes physician, pharmacy, and facility claims submitted for reimbursement for covered members. Both paid and denied claims are included in the database and analysis, except for pharmacy claims where only paid claims are included in the analysis. The EHR component includes clinical diagnoses, procedures, prescriptions, clinical notes, laboratory results, and vital signs (including BMI) recorded as part of routine clinical practice.

### Study populations

We created 3 nested study cohorts using different components of OLDW to evaluate the availability (Cohort 1) and validity (Cohorts 2 and 3) of claims-based weight-related diagnosis codes before and after the bariatric surgical operation (Additional file 1 eFigure 1). The study was approved by the Harvard Pilgrim Health Care institutional review board with an exemption and waiver of individual patient consent.

#### Cohort 1

Using the claims data, we identified a retrospective cohort of patients aged 18 years or older who underwent adjusted gastric banding (AGB), Roux-en-Y gastric bypass (RYGB), or sleeve gastrectomy (SG) between January 1, 2011 and June 30, 2018. Eligible patients had continuous health plan enrollment with medical and pharmacy benefits during the 6-month period preceding the index bariatric operation, which could occur in an inpatient or ambulatory care setting. To minimize the inclusion of patients with non-obesity indications, we excluded patients who had any major bariatric operation, revisional procedures, or gastrointestinal malignancy in the 6-month preoperative period, as well as patients who had an emergency department encounter or a diagnosis of gastrointestinal ulcers on the day of the index operation. We further excluded patients who had multiple conflicting bariatric operation procedure codes on the day of index operation. The cohort was identified using ICD-9-CM (prior to October 1, 2015) and ICD-10-CM (on or after October 1, 2015) diagnosis and procedure codes; Current Procedural Terminology, Fourth Edition (CPT-4®); and the Healthcare Common Procedure Coding System. We used this cohort to evaluate the availability of claims-based weight-related diagnosis codes before and after the bariatric operation.

#### Cohorts 2 and 3

Cohort 2 consisted of the subset of patients in Cohort 1 who had ≥1 preoperative claims-based weight-related diagnosis code with the last available code being *granular* (e.g., V85.30 or Z68.30 indicating BMI between 30.0–30.9 kg/m^2^) and ≥ 1 EHR-based BMI measurement recorded ±30 days of the granular code during the 6-month preoperative period (including the index operation day). We used this cohort to evaluate the performance of our claims-based severe obesity and BMI categorization algorithms (defined below) in the preoperative period. Cohort 3 consisted of the subset of patients in Cohort 2 whose last available claims-based postoperative weight-related diagnosis was a granular code with ≥1 EHR-based BMI measurement recorded ±30 days of this diagnosis code during the 1-year postoperative period. We used Cohort 3 to evaluate the performance of our claims-based algorithms in the postoperative period.

### Development of claims-based algorithms for severe obesity and BMI categorization

We created 2 claims-based algorithms using weight-related diagnosis codes (Additional file 1 eTable 1): a *severe obesity classification algorithm* and a *BMI categorization algorithm*. The severe obesity classification algorithm classified patients as having “severe obesity” if they had ≥1 claims-based weight-related diagnosis code indicating BMI ≥35 kg/m^2^ any time during the 6-month preoperative period. In bariatric surgery research, this algorithm can be used as an important cohort selection criterion to identify patients with severe obesity as the treatment indication.

The BMI categorization algorithm classified a patient’s BMI into 1 of the 10 levels as indicated by their last available weight-related diagnosis codes separately during the 6-month preoperative and 1-year postoperative periods (BMI levels, kg/m^2^: ≤19.9, 20.0–24.9, 25.0–29.9, 30.0–34.9, 35.0–39.9, 40.0–44.9, 45.0–49.9, 50.0–59.9, 60.0–69.9, and ≥ 70.0). This algorithm can be used to measure the last available preoperative BMI, which is an important covariate for comparative effectiveness research on bariatric surgery as preoperative BMI may be associated both with operation choice and risks of many health outcomes. The algorithm can also measure the last available BMI measurement within a defined postoperative follow-up period (e.g., 1 year in this study) for weight-related outcome assessment.

### Validation of claims-based algorithms for severe obesity and BMI categorization

We used the EHR-based BMI measurements recorded during an encounter to validate the claims-based algorithms. We classified patients as having severe obesity if they had ≥1 EHR-based BMI measurements ≥35 kg/m^2^ any time during the 6-month preoperative period. For BMI categorization, we classified a patient’s most proximate EHR-based BMI measurement recorded ±30 days of the last available claims-based diagnosis code in the 6-month preoperative period (for preoperative analyses) and the last available EHR-based BMI measurement in the 1-year postoperative period (for postoperative analyses), separately, into 1 of the 10 levels described above.

### Statistical analyses

#### Availability and predictors of weight-related diagnosis codes during the preoperative and postoperative periods

We described the presence of weight-related ICD-9-CM and ICD-10-CM diagnosis codes occurring any time in the 6-month preoperative period and the 1-year postoperative period, separately, in Cohort 1. We also performed the analysis by operation type, calendar year, and coding era (before October 1, 2015 for the ICD-9-CM era; October 1, 2015 and later for the ICD-10-CM era). We assessed factors associated with the presence of preoperative and postoperative claims-based weight-related diagnosis codes, separately, using logistic regression models. Factors selected a priori included demographic characteristics, region of residence, calendar year, coding era, type of index bariatric operation, care setting of index operation, and medical history measured in the 6-month preoperative period (including the Charlson-Elixhauser comorbidity index score [15], individual comorbid conditions, and prior hospital admissions). The Charlson-Elixhauser comorbidity index score was originally developed to predict mortality risk in older patients [15]; we used the score as a proxy for general health status.

#### Performance of the severe obesity classification algorithm during the preoperative period

We assessed the performance of the severe obesity classification algorithm using sensitivity, specificity, positive predictive value (PPV), and negative predictive value (NPV) within Cohort 2. The sensitivity was calculated as the proportion of patients accurately classified as having severe obesity based on claims-based diagnosis code (i.e., true positives) among those classified as such based on their EHR-based BMI measurement. The specificity was calculated as the proportion of patients accurately classified as not having severe obesity based on claims-based diagnosis codes (i.e., true negatives) among those whose EHR-based BMI measurement indicated as such. The PPV was calculated as the proportion of true positives among patients classified as having severe obesity based on their claims-based diagnosis code. The NPV was calculated as the proportion of true negatives among patients classified as not having severe obesity based on diagnosis code.

#### Performance of the BMI categorization algorithm during the preoperative and postoperative periods

We evaluated the performance of the BMI categorization algorithm separately in the 6-month preoperative period using Cohort 2 and in the 1-year postoperative period using Cohort 3. In both preoperative and postoperative periods, we assessed the concordance between the last available claims-based weight-related diagnosis code and its most proximate EHR-based BMI measurement recorded ±30 days of the claims-based diagnosis code by estimating the weighted Cohen’s kappa. As a variation of the Cohen’s kappa, a measure of the degree of agreement, the weighted kappa assigns weights for partial agreement according to their distance from the perfect agreement [16]. The weighted kappa ranges from − 1 to 1 with negative values possible but unlikely in practice. In general, kappa values >0.75 are considered excellent, 0.45–0.75 are considered fair to good, and <0.40 are considered poor agreement [17]. In both preoperative and postoperative periods, we also estimated the sensitivity, specificity, PPV, and NPV within each level of the algorithm.

#### Sensitivity analyses

We examined a different severe obesity classification algorithm using BMI ≥40 kg/m^2^ as the cutoff. We also varied the BMI categorization algorithm by (1) using larger BMI intervals (5-level BMI categories, kg/m^2^: ≤29.9, 30.0–39.9, 40.0–49.9, 50.0–59.9, ≥60.0; 4-level categories: underweight ≤19.9, normal 20.0–24.9, overweight 25.0–29.9, obese ≥30.0), and (2) adding nonspecific weight-related diagnosis codes (e.g., 278.00/E66.9 [unspecific obesity], 278.01/E66.01 [morbid obesity], 278.03/E66.2 [obesity hypoventilation syndrome], E66.09 [other obesity due to excess calories], E66.1 [drug-induced obesity], and E66.8 [other obesity] for obese) and assessed their performance during the preoperative and postoperative periods (Additional file 1 eTable 2). In addition, we examined the impact of the proximity restriction between the claims-based weight-related diagnosis code and the EHR-based BMI measurement on their concordance in the preoperative and postoperative periods. We also separately evaluated the performance of the BMI categorization algorithm for the last available BMI during the 6-month and 2-year postoperative periods. We performed all analyses with SAS Enterprise Guide 7.13 for Windows (SAS Institute, Cary, NC).

## Results

### Population characteristics

Cohort 1 included 29,357 patients, with 2941 (10.0%) having AGB, 9445 (32.2%) having RYGB, and 16,971 (57.8%) having SG. Table 1 shows their baseline characteristics. The population was largely female (75.5%) and white (67.0%) with a mean age of 47.0 years. The most prevalent comorbid conditions were hypertension (68.8%), gastroesophageal reflux disease (62.7%), and dyslipidemia (55.3%).

**Table 1:** Baseline characteristics of 29,357 patients who received a bariatric surgical operation, 2011–2018 (Cohort 1)

Characteristics	Overall		AGB		RYGB		SG	
	N	%	N	%	N	%	N	%
N (%)	29,357	100	2941	10.0	9445	32.2	16,971	57.8
Age, year								
Mean (SD)	47.0	12.3	45.6	12.4	48.7	12.4	46.3	12.1
Median (IQR)	47	38–56	45	36–55	49	40–58	46	37–55
18–44	12,857	43.8	1444	49.1	3596	38.1	7817	46.1
45–64	13,898	47.3	1256	42.7	4817	51.0	7825	46.1
≥ 65	2602	8.9	241	8.2	1032	10.9	1329	7.8
Female sex	22,171	75.5	2248	76.4	7108	75.3	12,815	75.5
Race/ethnicity								
Asian	367	1.3	41	1.4	118	1.2	208	1.2
Black	4960	16.9	493	16.8	1495	15.8	2972	17.5
Hispanic	3484	11.9	367	12.5	1015	10.7	2102	12.4
White	19,661	67.0	1972	67.1	6548	69.3	11,141	65.6
Missing	885	3.0	68	2.3	269	2.8	548	3.2
Commercial insurance	23,428	79.8	2508	85.3	6967	73.8	13,953	82.2
Region								
Northeast	3545	12.1	386	13.1	1013	10.7	2146	12.6
Midwest	6491	22.1	506	17.2	2523	26.7	3462	20.4
South	15,391	52.4	1678	57.1	4572	48.4	9141	53.9
West	3930	13.4	371	12.6	1337	14.2	2222	13.1
Year of index operation								
2011	4267	14.5	1286	43.7	1849	19.6	1132	6.7
2012	3351	11.4	690	23.5	1349	14.3	1312	7.7
2013	3816	13.0	428	14.6	1284	13.6	2104	12.4
2014	3605	12.3	255	8.7	1089	11.5	2261	13.3
2015	3761	12.8	134	4.6	1060	11.2	2567	15.1
2016	4088	13.9	81	2.8	1081	11.4	2926	17.2
2017	4493	15.3	50	1.7	1169	12.4	3274	19.3
2018	1976	6.7	17	0.6	564	6.0	1395	8.2
Last BMI before operation, kg/m^2 a^								
Mean (SD)	46.1	9.0	44.2	7.7	46.2	9.8	46.3	8.6
Median (IQR)	44.8	40.4–50.9	42.6	39–47.5	45.6	40.6–51.8	44.5	40.6–50.7
Missing, %	25,026	85.2	2633	89.5	7918	83.8	14,475	85.3
Charlson-Elixhauser comorbidity score								
Mean (SD)	1.0	1.8	0.5	1.4	1.2	2.0	0.9	1.7
Median (IQR)	1	0–2	0	0–1	1	0–2	1	0–2
≤ −1	4928	16.8	704	23.9	1401	14.8	2823	16.6
0	9134	31.1	1128	38.4	2656	28.1	5350	31.5
≥ 1	15,295	52.1	1109	37.7	5388	57.0	8798	51.8
Number of inpatient admissions during the 6 months before operation, mean (SD)	0.1	0.3	0.0	0.2	0.1	0.4	0.0	0.3
Number of inpatient hospital days in the 6 months before operation, mean (SD)	0.3	2.2	0.2	1.1	0.5	3.2	0.2	1.7
Number of inpatient hospital days for index operation, mean (SD)	2.8	2.9	0.4	0.9	3.7	3.3	2.7	2.6
Health conditions								
Anxiety	9637	32.8	731	24.9	3180	33.7	5726	33.7
Deep vein thrombosis	380	1.3	22	0.7	142	1.5	216	1.3
Depression	9159	31.2	770	26.2	3269	34.6	5120	30.2
Dyslipidemia	16,232	55.3	1482	50.4	5670	60.0	9080	53.5
Eating disorder	3373	11.5	270	9.2	1212	12.8	1891	11.1
Diabetes	10,229	34.8	727	24.7	4177	44.2	5325	31.4
Hypertension	20,184	68.8	1853	63.0	6913	73.2	11,418	67.3
GERD	18,412	62.7	1607	54.6	6160	65.2	10,645	62.7
Infertility	182	0.6	13	0.4	56	0.6	113	0.7
Kidney diseases	2013	6.9	107	3.6	864	9.1	1042	6.1
NAFLD	5620	19.1	374	12.7	2069	21.9	3177	18.7
Osteoarthritis, lower limb	2262	7.7	147	5.0	739	7.8	1376	8.1
PCOS	1462	5.0	131	4.5	452	4.8	879	5.2
Psychotic disorder	1552	5.3	119	4.0	580	6.1	853	5.0
Pulmonary embolism	413	1.4	20	0.7	149	1.6	244	1.4
Substance use disorder	1388	4.7	61	2.1	445	4.7	882	5.2
Sleep apnea	16,071	54.7	1247	42.4	5448	57.7	9376	55.2
Smoker	1241	4.2	167	5.7	458	4.8	616	3.6

*Abbreviations*: *BMI* body mass index, *GERD* gastroesophageal reflux disease, *IQR* interquartile range, *NAFLD* non-alcoholic fatty liver disease, *PCOS* polycystic ovarian syndrome, *SD* standard deviation

^a^ The last BMI before operation was obtained in the electronic health records (EHR) component of the OptumLabs Data Warehouse (OLDW) for those patients who had linkage. Patients who did not have EHR linkage in the OLDW were coded as missing

Cohort 2 included 3045 patients from Cohort 1 who had both claim-based weight-related diagnosis codes (with the last preoperative code being granular) and EHR-based BMI measurements in the 6-month preoperative period; 196 (6.4%) had AGB, 1251 (34.6%) had RYGB, and 1794 (58.9%) had SG. Compared to Cohort 1, the average age was slightly higher (47.6 years), and slightly more patients had hypertension (69.5%) and dyslipidemia (56.4%) in Cohort 2. On the index operation day, 77.6% had both claims-based diagnosis codes and EHR-based BMI measurements.

Cohort 3 included 511 patients from Cohort 2 who had granular last available claims-based weight-related diagnosis codes in the 1-year postoperative period with ≥1 EHR-based BMI measurement in the ±30 days of the diagnosis code, with 31 (6.1%) having AGB, 190 (37.2%) having RYGB, and 290 (56.8%) having SG. Compared to Cohorts 1 and 2, the average age was higher (48.9 years) in Cohort 3, more patients had hypertension (71.8%) and dyslipidemia (58.1%), and fewer had non-alcoholic fatty liver disease (22.5%) or diagnosis codes indicating smoking (1.8%). On average, patients had their first weight-related diagnosis code around 57 days after index operation and last available diagnosis code 159 days before the end of 1-year follow-up.

### Presence of weight-related diagnosis codes

#### 6-month preoperative period

**Fig. 1:**
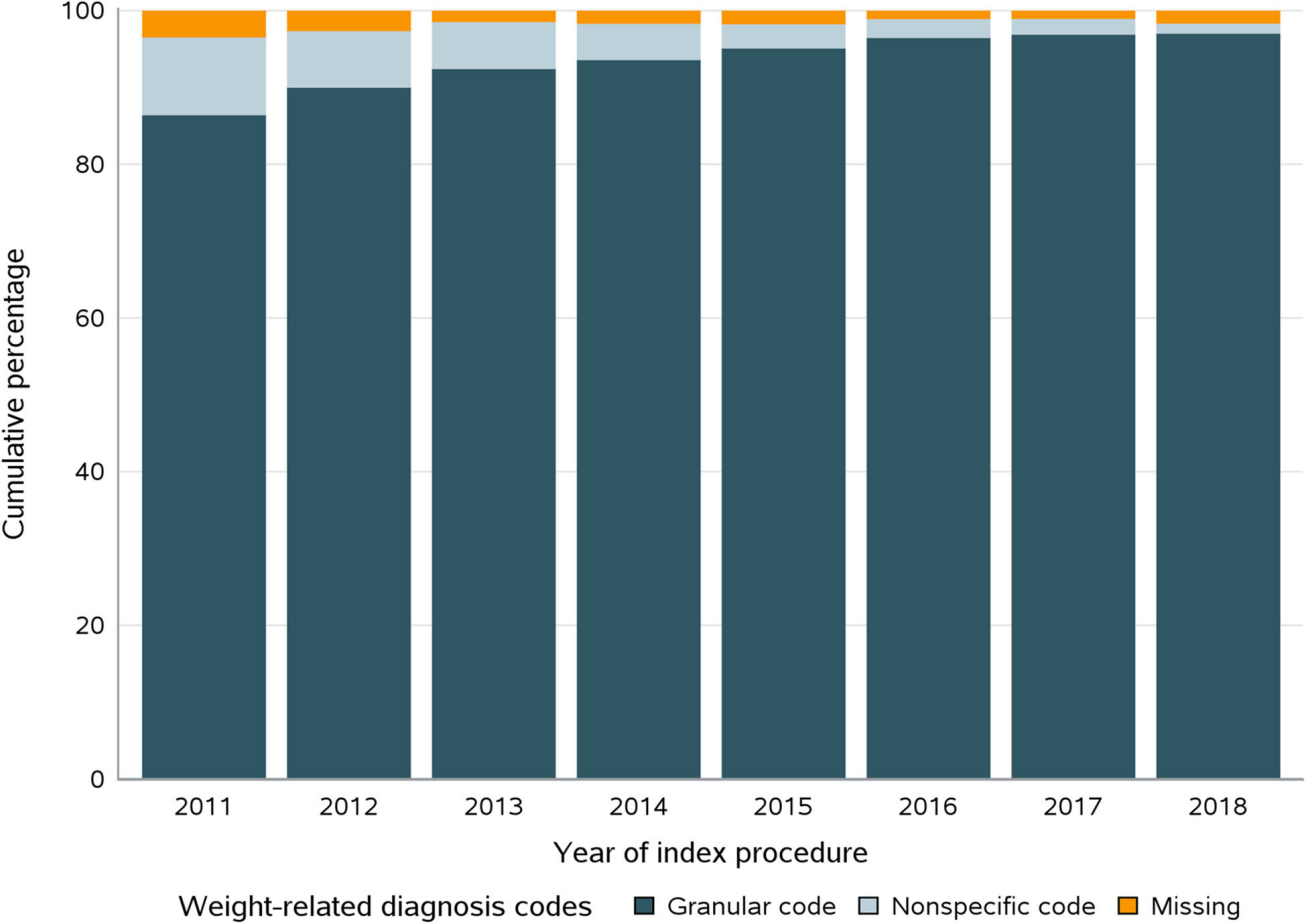
Presence of claims-based weight-related ICD-9-CM or ICD-10-CM diagnosis codes during the 6-month preoperative period for bariatric surgery patients in 2011–2018

Most of the patients in Cohort 1 had ≥1 claims-based weight-related diagnosis code, with 27,407 (93.4%) having granular codes, 1421 (4.8%) having nonspecific codes, and 529 (1.8%) having none. The prevalence of patients without a weight-related diagnosis code decreased from 3.4% in 2011 to 1.6% in 2018, while the presence of granular codes increased from 86.5% in 2011 to 97.1% in 2018 (Figure 1). The granular diagnosis codes were more prevalent in the ICD-10-CM era than the ICD-9-CM era (96.8% versus 91.1%). Similar increasing trends were observed across operation types, with higher prevalence of granular diagnosis codes observed in SG patients (Additional file 1 eFigures 2 & 3).

#### 1-year postoperative period

**Fig. 2:**
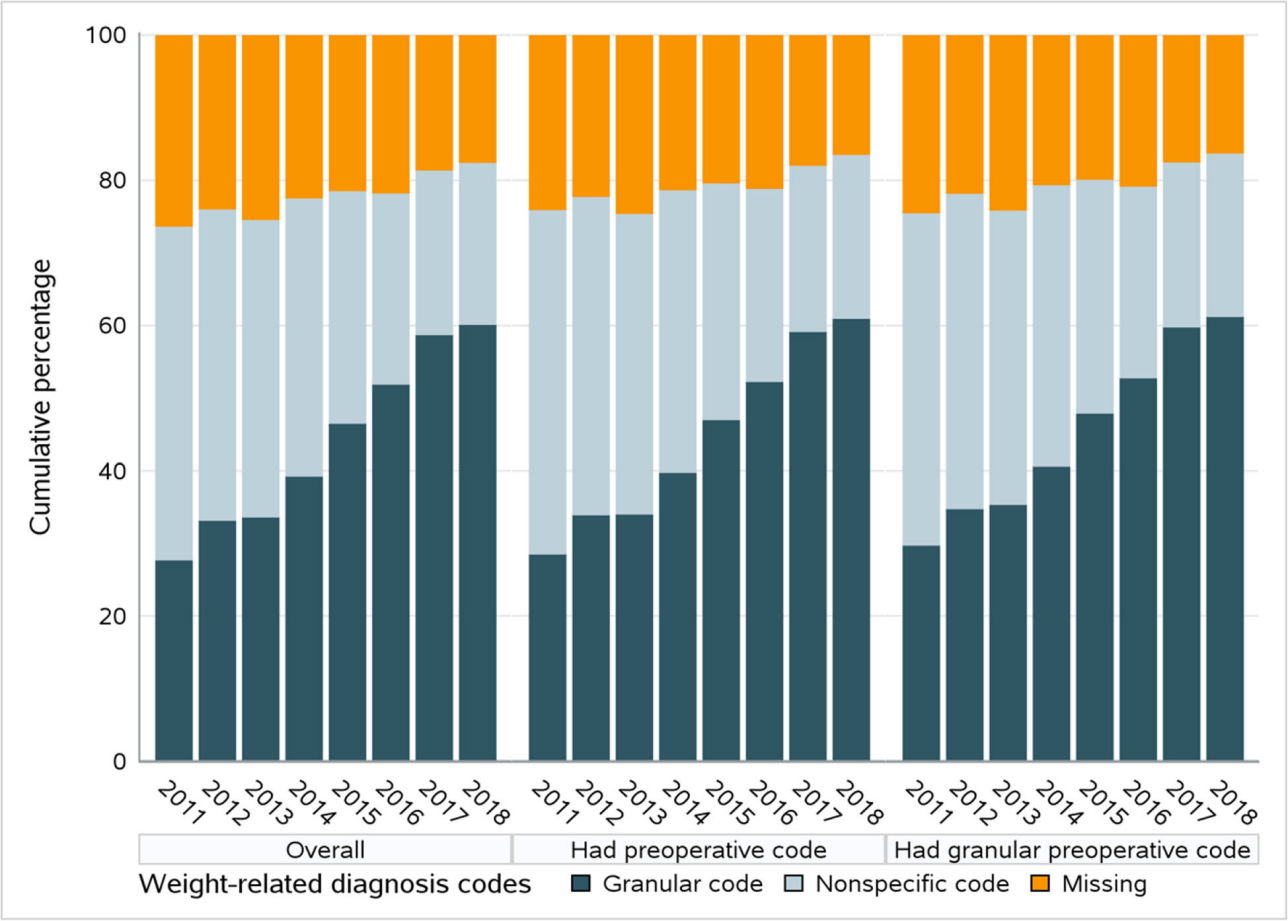
Presence of claims-based weight-related ICD-9-CM or ICD-10-CM diagnosis codes during the first postoperative year. Left panel: among all patients who underwent one of the three main bariatric surgical operations in 2011–2018; Middle panel: among patients who had weight-related diagnosis codes during the 6-month preoperative period; Right panel: among patients who had granular weight-related diagnosis codes during the 6-month preoperative period. Granular codes are diagnosis codes denoting narrow body mass index (BMI) ranges (e.g., V85.30 or Z68.30 indicating BMI between 30.0 and 30.9 kg/m^2^); Nonspecific codes are diagnosis codes denoting broad BMI ranges or obesity status

Among the 27,407 patients with granular weight-related diagnosis codes in the 6-month preoperative period in Cohort 1, 12,346 (45.0%) had granular codes, 9355 (34.1%) had nonspecific codes, and 5706 (20.8%) did not have any codes in the first postoperative year (Fig. 2). The distribution of diagnosis codes was similar among patients receiving different types of operation.

### Factors associated with the presence of weight-related diagnosis codes

#### 6-month preoperative period

Compared to patients with claims-based weight-related diagnosis codes, those without codes were more likely to be male, Asian, older, have more hospital stays before operation, or receive the operation in an ambulatory care setting in Cohort 1 (Table 2). Among patients who had weight-related diagnosis codes, those with granular codes (e.g., V85.30) were more likely to have SG, be covered by Medicare Advantage plans, or have the operation in an inpatient setting or recent years (Additional file 1 eTable 3).

**Table 2:** Determinants of missing weight-related diagnosis codes during the 6-month preoperative period, 2011–2018 (Cohort 1)

Determinant	Odd ratio, 95% confidence interval	
	Unadjusted^a^	Multivariable Adjusted^b^
Type of index operation (reference SG)		
AGB	0.62 (0.38–1.03)	0.20 (0.11–0.34)
RYGB	4.18 (3.46–5.06)	2.95 (2.33–3.74)
Care setting of index operation (reference Outpatient)		
Inpatient	0.90 (0.73–1.11)	0.37 (0.27–0.50)
Year of index operation (reference 2011)		
2012	0.75 (0.57–0.98)	0.86 (0.61–1.21)
2013	0.41 (0.30–0.56)	0.47 (0.32–0.70)
2014	0.47 (0.35–0.64)	0.49 (0.33–0.71)
2015	0.49 (0.36–0.66)	0.57 (0.38–0.85)
2016	0.29 (0.21–0.41)	0.43 (0.19–0.99)
2017	0.29 (0.20–0.40)	0.34 (0.15–0.78)
2018	0.46 (0.32–0.68)	0.68 (0.29–1.61)
ICD-CM coding era (reference ICD-10-CM)^c^		
ICD-9-CM	2.00 (1.64–2.44)	1.44 (0.71–2.90)
Age at index operation (reference 18–44 y)		
45–64	2.18 (1.71–2.79)	3.54 (2.65–4.72)
65+	13.28 (10.37–17.01)	25.63 (17.11–38.39)
Sex (reference male)		
Female	0.46 (0.38–0.55)	0.41 (0.32–0.52)
Race/ethnicity (reference White)		
Asian	3.79 (2.49–5.76)	3.98 (2.19–7.22)
Black	0.74 (0.57–0.96)	0.91 (0.66–1.25)
Hispanic	0.69 (0.51–0.94)	0.99 (0.68–1.44)
Unknown	0.95 (0.58–1.58)	1.14 (0.62–2.10)
Region of residence (reference northeast)		
Midwest	1.70 (1.21–2.39)	1.46 (0.96–2.24)
South	1.40 (1.02–1.92)	1.57 (1.05–2.36)
West	1.47 (1.01–2.14)	1.39 (0.87–2.22)
Type of insurance (reference Medicare Advantage)		
Commercial	0.38 (0.32–0.45)	0.93 (0.68–1.29)
Charlson-Elixhauser comorbidity score (reference ≤ − 1)		
0	2.22 (1.42–3.47)	2.52 (1.53–4.13)
1+	5.59 (3.70–8.44)	6.11 (3.79–9.86)
Number of hospital stays in the last 6 months (reference 0)		
1	7.54 (5.98–9.51)	10.39 (3.97–27.21)
2+	23.15 (17.06–31.40)	13.18 (6.12–28.37)
Length of hospital stays in the last 6 months (reference 0)		
1–4	4.70 (3.39–6.53)	0.28 (0.09–0.91)
5+	17.49 (13.96–21.93)	--^d^
Comorbid conditions		
Diabetes	0.53 (0.38–0.73)	0.25 (0.17–0.37)
Hypertension	0.58 (0.48–0.68)	0.49 (0.38–0.64)
GERD	0.58 (0.49–0.69)	0.81 (0.65–1.00)
NAFLD	0.38 (0.28–0.52)	0.35 (0.25–0.50)
PCOS	--^e^	0.10 (0.01–0.69)
PE	2.84 (1.80–4.49)	1.63 (0.86–3.09)
Anxiety	0.47 (0.38–0.59)	0.72 (0.55–0.94)
DVT	3.63 (2.36–5.58)	1.18 (0.61–2.30)
Depression	0.47 (0.38–0.59)	0.56 (0.43–0.74)
Dyslipidemia	0.61 (0.51–0.72)	0.46 (0.36–0.58)
Eating disorder	--^e^	0.07 (0.02–0.21)
Infertility	--^e^	--^e^
Kidney disease	4.61 (3.76–5.66)	1.92 (1.41–2.63)
Osteoarthritis	0.42 (0.26–0.67)	0.38 (0.22–0.65)
Psychotic disorder	0.70 (0.45–1.10)	0.60 (0.35–1.03)
Substance use disorder	1.95 (1.43–2.65)	2.13 (1.39–3.27)
Sleep apnea	0.07 (0.05–0.09)	0.06 (0.04–0.09)
Smoker	2.15 (1.58–2.93)	1.64 (1.08–2.50)

*Abbreviations*: *AGB* adjusted gastric banding, *DVT* deep vein thrombosis, *GERD* gastroesophageal reflux disease, *NAFLD* non-alcoholic fatty liver disease, *PCOS* polycystic ovarian syndrome, *PE* pulmonary embolism, *RYGB* Roux-en-Y gastric bypass, *SG* sleeve gastrectomy

^a^ Logistic regression models included only the variables under consideration

^b^ Logistic regression models adjusted for all variables listed in table

^c^ The ICD-9-CM era refers to the period prior to October 1, 2015, and the ICD-10-CM era refers to the period starting from October 1, 2015

^d^ The variable was removed from the model because it was as a linear combination of other variables included

^e^ Estimates are not provided because cells with 10 or fewer patients have been suppressed to maintain the de-identification nature of the database

#### 1-year postoperative period

Compared to patients with claims-based weight-related diagnosis codes, those without codes were more likely to receive AGB, be younger, be male, be commercially insured, or lack preoperative weight-related diagnosis codes in Cohort 1 (Additional file 1 eTable 4). Among patients who had weight-related diagnosis codes in the postoperative year, those having granular codes were more likely to be older, be covered by Medicare Advantage plans, have comorbid conditions, receive SG, or have the operation in an inpatient setting or recent years (Additional file 1 eTable 5).

### Performance of the claims-based algorithms

#### 6-month preoperative period

In Cohort 2, the severe obesity classification algorithm (i.e., presence of BMI ≥35 kg/m^2^) in the 6-month preoperative period had a sensitivity of 100%, a specificity of 71%, a PPV of 100%, and an NPV of 78% (Additional file 1 eTable 6). When classifying the last available preoperative weight-related diagnosis code into 10 levels, the BMI categorization algorithm had a weighted kappa of 0.78 (95% confidence interval 0.76, 0.79). The specificity and NPV were high for all BMI levels; The sensitivity and PPV were above 60% for most BMI levels over 35 kg/m^2^ (e.g., BMI 35.0–39.9, sensitivity 64%, specificity 97%, PPV 81%, NPV 93%; 40.0–44.9, sensitivity 76%, specificity 87%, PPV 71%, NPV 90%) and lowest for BMI between 30.0 and 34.9 kg/m^2^ (sensitivity 30%) (Table 3).

**Table 3:** Validation results for the BMI categorization algorithm in the 6-month preoperative (Cohort 2) and 1-year postoperative periods (Cohort 3)

BMI categories, kg/m^**2**^	ICD-9-CM or ICD-10-CM Diagnoses	Last preoperative BMI^**a**^					Last postoperative BMI^**b**^				
		Sensitivity	Specificity	PPV	NPV	Kappa (95% CI)	Sensitivity	Specificity	PPV	NPV	Kappa (95% CI)
≤19.9	V85.0, Z68.1	100	100	80	100	0.78 (0.76, 0.79)	100	100	100	100	0.84 (0.80,0.87)
20.0–24.9	V85.1, Z68.20-Z68.24	60	100	100	100		75	100	100	100	
25.0–29.9	V85.21-V85.25, Z68.25-Z68.29	70	100	100	100		> 82^c^	> 98^c^	> 82^c^	98	
30.0–34.9	V85.30-V85.34, Z68.30-Z68.34	30	100	> 62^c^	99		84	> 97^c^	> 93^c^	93	
35.0–39.9	V85.35-V85.39, Z68.35-Z68.39	64	97	81	93		84	94	81	95	
40.0–44.9	V85.41, Z68.41	76	87	71	90		76	93	68	95	
45.0–49.9	V85.42, Z68.42	71	90	65	92		72	97	73	97	
50.0–59.9	V85.43, Z68.43	79	93	76	94		> 65^c^	97	61	> 98^c^	
60.0–69.9	V85.44, Z68.44	70	98	67	98		100	99	17	100	
≥70.0	V85.45, Z68.45	53	100	63	99		--^d^	100	0	100	
≤29.9	V85.0, V85.1, V85.21-V85.25, Z68.1, Z68.20-Z68.29	87	100	100	100	0.77 (0.76, 0.79)	> 84^c^	> 98^c^	> 84^c^	98	0.82 (0.78, 0.87)
30.0–39.9	V85.30-V85.39, Z68.30-Z68.39	67	98	90	93		89	93	94	87	
40.0–49.9	V85.41, V85.42, Z68.41, Z68.42	89	81	82	88		86	93	80	95	
50.0–59.9	V85.43, Z68.43	79	93	76	94		> 65^c^	97	61	> 98^c^	
≥60.0	V85.44, V85.45, Z68.44, Z68.45	75	98	74	98		100	99	14	100	
≤19.9	V85.0, Z68.1	100	100	80	100	0.94 (0.89, 0.99)	100	100	100	100	0.90 (0.84, 0.96)
20.0–24.9	V85.1, Z68.20-Z68.24	60	100	100	100		75	100	100	100	
25.0–29.9	V85.21-V85.25, Z68.25-Z68.29	70	100	100	100		> 82^c^	> 98^c^	> 82^c^	98	
≥30.0	V85.30-V85.39, V85.41-V85.45, Z68.30-Z68.39, Z68.41-Z68.45	100	87	100	100		> 98^c^	> 81^c^	98	> 81^c^	

*Abbreviations*: *BMI* body mass index, *ICD* international classification of diseases, *PPV* positive predictive value, *NPV* negative predictive value, *CI* confidence interval

^a^ The last available weight-related diagnosis code in claims in the 6-month preoperative period was compared with the last available BMI measurement in the electronic health records (EHR) during the same period in Cohort 2 patients

^b^ The last available weight-related diagnosis code in claims in the 1-year postoperative period was compared with the last available BMI measurement in the EHR during the same period in Cohort 3 patients

^c^ Cells with 10 or fewer patients have been suppressed to maintain the de-identification nature of the database

^d^ Sensitivity was not calculated because no patients had relevant BMI measurement at this level in the EHR

#### 1-year postoperative period

In Cohort 3, the BMI categorization algorithm had a weighted kappa of 0.84 (95% confidence interval 0.80, 0.87). The specificity and NPV were high for all BMI levels while the sensitivity was above 70% and the PPV was above 60% for most BMI levels (Table 3).

#### Sensitivity analyses

When varying the severe obesity classification algorithm to detect the presence of BMI ≥40 kg/m^2^ during the 6-month preoperative period, both the specificity and NPV increased (75 and 83%, respectively) while sensitivity and PPV dropped slightly (98 and 96%, respectively). Expanding the algorithms to include nonspecific weight-related diagnosis codes (e.g., 278.01) resulted in meaningful decrease in specificity (Additional file 1 eTable 6).

The 5-level BMI categorization algorithm had similar concordance compared to the 10-level categorization, while the 4-level BMI categorization algorithm had great concordance with a weighted kappa above 0.90 for both the preoperative and postoperative periods (Table 3). Expanding the algorithms to include nonspecific weight-related diagnosis codes had minimal impact on their performance (Additional file 1 eTable 7). Relaxing the proximity requirement between the timing of the claims-based weight-related diagnosis codes and the EHR-based BMI measurements increased the size of the validation sample; this did not change their concordance during the 6-month preoperative period but reduced their concordance in the 1-year postoperative period (Additional file 1 eFigure 4). The BMI categorization algorithm for the last available BMI performed well in the 6-month and 2-year postoperative periods (Additional file 1 eTable 8).

## Discussion

In a large administrative claims database, we found that nearly all bariatric surgery patients had preoperative weight-related diagnosis codes, while the presence of granular weight-related diagnosis codes increased substantially in both the preoperative and postoperative periods between 2011 and 2018. The claim-based algorithm for severe obesity, which classified patients as having severe obesity if they had a diagnosis code indicating BMI ≥35 kg/m^2^, had high sensitivity and PPV but reasonable specificity and NPV. The BMI categorization algorithm that categorized weight-related diagnosis codes into BMI levels had excellent concordance with the EHR-based BMI measurement, with high specificity, PPV, and NPV across all levels and higher sensitivity among higher levels of BMI.

The persistently high prevalence of claims-based weight-related diagnosis codes, including granular and nonspecific codes, in the preoperative period across the study years reflects the high adherence to the insurance reimbursement requirement [11–13]. The observed higher prevalence of weight-related diagnosis codes in the ICD-10-CM era than the ICD-9-CM era is consistent with previous data that focused on the claim-based diagnosis codes in the general population [10].

The BMI categorization algorithm had different sensitivities for BMI level 30.0–34.9 kg/m^2^ in the preoperative and postoperative periods (30% versus 84%). Six months before having a bariatric operation, 70% of patients with an EHR-based BMI measurement between 30.0 and 34.9 kg/m^2^ had a granular weight-related diagnosis code indicating BMI ≥35 kg/m^2^. During the first postoperative year, only 15% of those with an BMI measurement between 30.0 and 34.9 kg/m^2^ had a diagnosis code indicating BMI ≥35 kg/m^2^. These patients with borderline BMI levels immediately before having a bariatric operation might have undergone preoperative weight loss as required by their insurance or encouraged by their clinical programs, as half of them had 1 or more BMI measurements ≥35 kg/m^2^ within the prior 30 days. These patients might also have been up-coded with a higher weight-related diagnosis code to meet the prior authorization requirement.

### Claims databases for bariatric Surgery research: a glass half-full of half-empty?

The high prevalence and validity of weight-related diagnosis codes before a bariatric operation in claims databases makes it feasible to use these codes to capture a large proportion of eligible patients, especially when researchers impose additional eligibility criteria to exclude patients with non-obesity indications, like what we did in our study. In addition, the high concordance between the claims-based BMI categorization algorithm and actual BMI measurement, along with its high validity, suggests that it is possible to use these preoperative weight-related diagnosis codes for baseline confounding control.

On the other hand, despite considerable increase across years and high validity, the presence of weight-related diagnosis codes remained low in the first postoperative year, with around 80% of patients having any codes and around 60% having granular codes in 2017 and 2018. The suboptimal presence of weight-related diagnosis codes in the postoperative period makes it more challenging to use claims databases for weight-related effectiveness research. In addition, there could be differential coding in the postoperative period because patients with granular weight-related diagnosis codes were older and had more comorbid conditions (Additional file 1 eTable 5). These patients with granular diagnosis codes in the postoperative period may not be representative of the overall study population. For example, some of them may be preparing for a second stage operation or having inadequate weight loss from their index operation. It is thus important to weigh the internal validity and generalizability when using the postoperative weight-related diagnosis codes for weight-related effectiveness outcome research. In situations when all relevant factors contributing to the presence of postoperative granular diagnosis codes are measured, results from patients with granular codes could be generalized to the overall study population using appropriate statistical approaches, such as inverse probability weighting [18]. Taken together, our findings support the use of administrative claims data for bariatric surgery research of non-weight-related outcomes that are generally well-captured, such as rehospitalization, reoperation, venous thromboembolism, or remission of certain comorbidities including type 2 diabetes [19–22].

### Strengths and limitations

This study used contemporary data from a large administrative claim database linked with EHR to validate two claims-based weight-related algorithms. Prior studies focused on either claims-based algorithms in the general population [8, 10] or the broad four-level obesity classification algorithm for bariatric surgery patients in the preoperative period [23]. We evaluated the validity of these diagnosis codes during both the preoperative and postoperative periods, providing information for researchers who are interested in using administrative claims databases to study weight-related effectiveness outcomes. Our findings add to the knowledge base of the quality and suitability of administrative claims data, a real-world data source, for generation of real-world evidence in bariatric surgery research [24].

One limitation of our study is the small sample size for the postoperative period resulted from the proximity requirement on the EHR-based BMI measurement, which may limit the generalizability of our results. In sensitivity analyses where we relaxed the proximity requirement, the size of the validation sample increased but no substantial change was observed in the validity of postoperative weight-related diagnosis codes. Moreover, the linked EHR data were only available on a small subset of patients identified in claims who received care at healthcare service systems that contribute EHR data to OLDW, raising the possibility of unmeasured factors affecting our analyses and limiting the generalizability of our results.

## Conclusions

Among bariatric surgery patients identified within administrative claims databases, the validity of weight-related diagnosis codes was excellent during the preoperative and postoperative periods. These findings support the use of administrative claims databases for bariatric surgery research in the absence of BMI measurements for non-weight-related effectiveness and safety outcomes that are generally well-captured in these databases. However, the availability of weight-related diagnosis codes was suboptimal during the postoperative period, making it more challenging to use claims databases for weight-related effectiveness research.

## Supplementary information

**Additional file 1: eFigure 1.** Identification of eligible bariatric surgery patients in 3 nested cohorts for evaluation of availability and validity of weight-related diagnosis codes in both the preoperative and postoperative periods. **eFigure 2.** Presence of weight-related ICD-9-CM/ICD-10-CM diagnosis codes during the 6-month preoperative period for patients who underwent 1 of the 3 bariatric surgical operations in 2011–2018. **eFigure 3.** Presence of weight-related ICD-9-CM/ICD-10-CM diagnosis codes during the 6-month preoperative period for patients who underwent 1 of the 3 bariatric surgical operations in the ICD-9-CM and ICD-10-CM coding era between 2011 and 2018. **eTable 1.** Definition of the claims-based algorithms to classify morbid obesity and categorize the body mass index using ICD-9-CM and ICD-10-CM diagnosis codes. **eTable 2.** Variation of the claims-based algorithms to classify morbid obesity and categorize the body mass index using ICD-9-CM and ICD-10-CM diagnosis codes. **eTable 3.** Determinants of having granular weight-related diagnosis codes during the 6-month preoperative period in bariatric surgery patients, 2011–2018. **eTable 4.** Determinants of missing weight-related diagnosis codes in the first postoperative year in bariatric surgery patients, 2011–2018. **eTable 5.** Determinants of having granular weight-related diagnosis codes in the first postoperative year in bariatric surgery patients, 2011–2018. **eTable 6.** Performance of the modified claims-based severe obesity classification algorithm in the 6-month preoperative period (Cohort 2). **eTable 7.** Performance of the modified claims-based body mass index categorization algorithm in the 6-month preoperative (Cohort 2) and 1-year postoperative periods (Cohort 3). **eFigure 4.** The sample size and estimated weighted kappa when relaxing the proximity restriction between the weight-related diagnosis codes and the body mass index (BMI) measurement in the electronic health records for categorization of the last available BMI in the first postoperative year with the BMI categorization algorithm. **eTable 8.** Performance of the claims-based body mass index (BMI) categorization algorithm for the last available BMI in different postoperative periods.

## Data Availability

The dataset from this study is held securely in a virtual workspace at OptumLabs. Data sharing agreements prohibit the authors from making the dataset publicly available. Access may be granted to those who meet pre-specified criteria for confidential access upon mutual agreements with OptumLabs. The analytic codes are available from the authors upon request, understanding that the programs may rely upon coding templates or macros that are unique to the data environment.

## Abbreviations

AGB: Adjusted gastric banding
BMI: Body mass index
CI: Confidence interval
CPT-4®: Current Procedural Terminology, Fourth Edition
DVT: Deep vein thrombosis
EHR: Electronic health records
GERD: Gastroesophageal reflux disease
ICD-9-CM: International Classification of Diseases, Ninth Revision, Clinical Modification
ICD-10-CM: International Classification of Diseases, Tenth Revision, Clinical Modification
IQR: Interquartile range
NAFLD: Non-alcoholic fatty liver disease
NPV: Negative predictive value
OLDW: OptumLabs® Data Warehouse
PCOS: Polycystic ovarian syndrome
PE: Pulmonary embolism
PPV: Positive predictive value
RYGB: Roux-en-Y gastric bypass
SD: Standard deviation
SG: Sleeve gastrectomy
