# Supplementary material for: Suitability of administrative claims databases for bariatric surgery research – is the glass half-full or half-empty?
Source: BMC Med Res Methodol. 2020 Sep 7;20:225. doi: 10.1186/s12874-020-01106-8 (PMC7487952; doi:10.1186/s12874-020-01106-8)
Supplement: Supplementary file 1 — Additional file 1: eFigure 1. Identification of eligible bariatric surgery patients in 3 nested cohorts for evaluation of availability and validity of weight-related diagnosis codes in both the preoperative and postoperative periods. eFigure 2. Presence of weight-related ICD-9-CM/ICD-10-CM diagnosis codes during the 6-month preoperative period for patients who underwent 1 of the 3 bariatric surgical operations in 2011–2018. eFigure 3. Presence of weight-related ICD-9-CM/ICD-10-CM diagnosis codes during the 6-month preoperative period for patients who underwent 1 of the 3 bariatric surgical operations in the ICD-9-CM and ICD-10-CM coding era between 2011 and 2018. eTable 1. Definition of the claims-based algorithms to classify morbid obesity and categorize the body mass index using ICD-9-CM and ICD-10-CM diagnosis codes. eTable 2. Variation of the claims-based algorithms to classify morbid obesity and categorize the body mass index using ICD-9-CM and ICD-10-CM diagnosis codes. eTable 3. Determinants of having granular weight-related diagnosis codes during the 6-month preoperative period in bariatric surgery patients, 2011–2018. eTable 4. Determinants of missing weight-related diagnosis codes in the first postoperative year in bariatric surgery patients, 2011–2018. eTable 5. Determinants of having granular weight-related diagnosis codes in the first postoperative year in bariatric surgery patients, 2011–2018. eTable 6. Performance of the modified claims-based severe obesity classification algorithm in the 6-month preoperative period (Cohort 2). eTable 7. Performance of the modified claims-based body mass index categorization algorithm in the 6-month preoperative (Cohort 2) and 1-year postoperative periods (Cohort 3). eFigure 4. The sample size and estimated weighted kappa when relaxing the proximity restriction between the weight-related diagnosis codes and the body mass index (BMI) measurement in the electronic health records for categorization of the last available [file 12874_2020_1106_MOESM1_ESM.docx]

**Additional File 1**

“Suitability of Administrative Claims Databases for Bariatric Surgery Research – Is the Glass Half-full or Half-empty?”

**Contents:**

**eFigure 1.** Identification of eligible bariatric surgery patients in 3 nested cohorts for evaluation of availability and validity of weight-related diagnosis codes in both the preoperative and postoperative periods

**eFigure 2.** Presence of weight-related ICD-9-CM/ICD-10-CM diagnosis codes during the 6-month preoperative period for patients who underwent 1 of the 3 bariatric surgical operations in 2011-2018

**eFigure 3.** Presence of weight-related ICD-9-CM/ICD-10-CM diagnosis codes during the 6-month preoperative period for patients who underwent 1 of the 3 bariatric surgical operations in the ICD-9-CM and ICD-10-CM coding era between 2011-2018

**eTable 1.** Definition of the claims-based algorithms to classify morbid obesity and categorize the body mass index using ICD-9-CM and ICD-10-CM diagnosis codes

**eTable 2.** Variation of the claims-based algorithms to classify morbid obesity and categorize the body mass index using ICD-9-CM and ICD-10-CM diagnosis codes

**eTable 3.** Determinants of having granular weight-related diagnosis codes during the 6-month preoperative period in bariatric surgery patients, 2011-2018

**eTable 4.** Determinants of missing weight-related diagnosis codes in the first postoperative year in bariatric surgery patients, 2011-2018

**eTable 5.** Determinants of having granular weight-related diagnosis codes in the first postoperative year in bariatric surgery patients, 2011-2018

**eTable 6.** Performance of the modified claims-based severe obesity classification algorithm in the 6-month preoperative period (Cohort 2)

**eTable 7.** Performance of the modified claims-based body mass index categorization algorithm in the 6-month preoperative (Cohort 2) and 1-year postoperative periods (Cohort 3)

**eFigure 4.** The sample size and estimated weighted kappa when relaxing the proximity restriction between the weight-related diagnosis codes and the body mass index (BMI) measurement in the electronic health records for categorization of the last available BMI in the first postoperative year with the 10-level BMI categorization algorithm

**eTable 8.** Performance of the claims-based body mass index (BMI) categorization algorithm for the last available BMI in different postoperative periods

During the 6-month baseline period:

• not continuously enrolled with medical and pharmacy benefits (N=4,613)

• having surgery or revisional codes (N=2,984)

• having gastrointestinal cancers (N=797)

On the day of index operation:

• having an emergency department encounter (N=1,038)

• aged <18 years (N=96)

• having multiple operation procedure codes (N=561)

During index operation:

• gastrointestinal ulcer on the day of the index operation (N=283)

• index hospitalization > 30 days (N=145)

• conflicting or missing demographic information (N=28)

Patients who underwent bariatric surgery during an inpatient admission or ambulatory care between 01/01/2011 and 6/30/2018

N=39,902

Eligible patients identified using claims (Cohort 1)

N=29,357

During the 6-month baseline period:

• no EHR linkage (N=18,526)

• no encounter information (N=193)

• no EHR-based BMI measurements (N=6,307)

• no claims-based weight-related diagnosis (N=79)

Eligible patients with baseline and follow-up claims-based weight-related diagnoses and EHR-based BMI measurements (Cohort 3)

N= 511

During the 12-month follow-up period

• no claims-based weight-related diagnosis code (N=521)

• last available weight-related diagnosis code was nonspecific (N=1,617)

• no EHR-based BMI measurement within 30 days of the last-available claims-based diagnosis code (N= 396)

During the 6-month baseline period:

• last available claims-based weight-related diagnosis code was nonspecific (N=216)

• no EHR-based BMI measurement within 30 days of the last-available claims-based diagnosis code (N=991)

Eligible patients with claims-based weight-related diagnoses and EHR-based BMI measurements in the 6-month preoperative period (Cohort 2)

N=3,045

**Evaluation of validity of weight-related diagnosis codes in the postoperative period**

**Evaluation of validity of weight-related diagnosis codes in the preoperative period**

**Evaluation of availability of weight-related diagnosis codes**

**eFigure 1.** Identification of eligible bariatric surgery patients in 3 nested cohorts for evaluation of availability and validity of weight-related diagnosis codes in both the preoperative and postoperative periods.

**Evaluation of validity of weight-related diagnosis codes in the preoperative period**

**Evaluation of availability of weight-related diagnosis codes**

*

*

*Categories with 10 or fewer patients in the AGB group (in 2012 and 2015-2017) and the SG group in (2016-2018) are suppressed to maintain the de-identification nature of the database.

**eFigure 2.** Presence of weight-related ICD-9-CM/ICD-10-CM diagnosis codes during the 6-month preoperative period for patients who underwent 1 of the 3 bariatric surgical operations in 2011-2018. AGB = adjusted gastric banding; RYGB = Roux-en-Y gastric bypass; SG = sleeve gastrectomy. Granular codes are diagnosis codes indicating narrow BMI ranges (e.g., V85.30 or Z68.30 indicating BMI of 30.0-30.9 kg/m^2^); Nonspecific codes are diagnosis codes denoting broad BMI ranges or obesity status.

*

*Groups with 10 or fewer patients in the AGB in the ICD-10-CM era are suppressed to maintain the de-identification nature of the database.

**eFigure 3.** Presence of weight-related ICD-9-CM/ICD-10-CM diagnosis codes during the 6-month preoperative period for patients who underwent 1 of the 3 bariatric surgical operations in the ICD-9-CM and ICD-10-CM coding era between 2011-2018. AGB = adjusted gastric banding; RYGB = Roux-en-Y gastric bypass; SG = sleeve gastrectomy. Granular codes are diagnosis codes denoting narrow BMI ranges (e.g., V85.30 or Z68.30 indicating BMI of 30.0-30.9 kg/m^2^); Nonspecific codes are diagnosis codes denoting broad BMI ranges or obesity status.

**eTable 1.** Definition of the claims-based algorithms to classify morbid obesity and categorize the body mass index (BMI, kg/m^2^) using ICD-9-CM and ICD-10-CM diagnosis codes

| **Algorithm** | **Classification** | **Weight-related diagnosis codes** | |
| --- | --- | --- | --- |
|  |  | **ICD-9-CM** | **ICD-10-CM** |
| Morbid obesity | Having ≥1 BMI measurement ≥35.0 kg/m^2^ | V85.35, V85.36, V85.37, V85.38, V85.39 | Z68.35, Z68.36, Z68.37, Z68.38, Z68.39 |
|  |  | V85.41, V85.42, V85.43, V85.44, V85.45 | Z68.41, Z68.42, Z68.43, Z68.44, Z68.45 |
| 10-level BMI categorization | ≤19.9 | V85.0 | Z68.1 |
|  | 20.0-24.9 | V85.1 | Z68.20, Z68.21, Z68.22, Z68.23, Z68.24 |
|  | 25.0-29.9 | V85.21, V85.22, V85.23, V85.24, V85.25 | Z68.25, Z68.26, Z68.27, Z68.28, Z68.29 |
|  | 30.0-34.9 | V85.30, V85.31, V85.32, V85.33, V85.34 | Z68.30, Z68.31, Z68.32, Z68.33, Z68.34 |
|  | 35.0-39.9 | V85.35, V85.36, V85.37, V85.38, V85.39 | Z68.35, Z68.36, Z68.37, Z68.38, Z68.39 |
|  | 40.0-44.9 | V85.41 | Z68.41 |
|  | 45.0-49.9 | V85.42 | Z68.42 |
|  | 50.0-59.9 | V85.43 | Z68.43 |
|  | 60.0-69.9 | V85.44 | Z68.44 |
|  | ≥70.0 | V85.45 | Z68.45 |
| 5-level BMI categorization | ≤29.9 | V85.0, V85.1, V85.21, V85.22, V85.23, V85.24, V85.25 | Z68.1, Z68.20, Z68.21, Z68.22, Z68.23, Z68.24 |
|  |  |  | Z68.25, Z68.26, Z68.27, Z68.28, Z68.29 |
|  | 30.0-39.9 | V85.30, V85.31, V85.32, V85.33, V85.34 | Z68.30, Z68.31, Z68.32, Z68.33, Z68.34 |
|  |  | V85.35, V85.36, V85.37, V85.38, V85.39 | Z68.35, Z68.36, Z68.37, Z68.38, Z68.39 |
|  | 40.0-49.9 | V85.41, V85.42 | Z68.41, Z68.42 |
|  | 50.0-59.9 | V85.43 | Z68.43 |
|  | ≥60.0 | V85.44, V85.45 | Z68.44, Z68.45 |
| 4-level BMI categorization | ≤19.9 (underweight) | V85.0 | Z68.1 |
|  | 20.0-24.9 (Normal weight) | V85.1 | Z68.20, Z68.21, Z68.22, Z68.23, Z68.24 |
|  | 25.0-29.9 (Overweight) | V85.21, V85.22, V85.23, V85.24, V85.25 | Z68.25, Z68.26, Z68.27, Z68.28, Z68.29 |
|  | ≥30.0 (Obese) | V85.30, V85.31, V85.32, V85.33, V85.34 | Z68.30, Z68.31, Z68.32, Z68.33, Z68.34 |
|  |  | V85.35, V85.36, V85.37, V85.38, V85.39 | Z68.35, Z68.36, Z68.37, Z68.38, Z68.39 |
|  |  | V85.41, V85.42, V85.43, V85.44, V85.45 | Z68.41, Z68.42, Z68.43, Z68.44, Z68.45 |

**eTable 2.** Variation of the claims-based algorithms to classify morbid obesity and categorize the body mass index (BMI, kg/m^2^) using ICD-9-CM and ICD-10-CM diagnosis codes

| **Algorithm** | **Classification** | **Weight-related diagnosis codes** | |
| --- | --- | --- | --- |
|  |  | **ICD-9-CM** | **ICD-10-CM** |
| Morbid obesity | Having ≥1 BMI measurement ≥35.0 kg/m^2^ | V85.35, V85.36, V85.37, V85.38, V85.39 | Z68.35, Z68.36, Z68.37, Z68.38, Z68.39 |
|  |  | V85.41, V85.42, V85.43, V85.44, V85.45 | Z68.41, Z68.42, Z68.43, Z68.44, Z68.45 |
|  |  | 278.01 | E66.01, E66.2 |
| Morbid obesity | Having ≥1 BMI measurement ≥40.0 kg/m^2^ | V85.41, V85.42, V85.43, V85.44, V85.45 | Z68.41, Z68.42, Z68.43, Z68.44, Z68.45 |
| Morbid obesity | Having ≥1 BMI measurement ≥40.0 kg/m^2^ | V85.41, V85.42, V85.43, V85.44, V85.45 | Z68.41, Z68.42, Z68.43, Z68.44, Z68.45 |
|  |  | 278.01 | E66.01, E66.2 |
| 10-level BMI categorization | ≤19.9 | 783.22, V85.0 | R63.6, Z68.1 |
|  | 20.0-24.9 | V85.1 | Z68.20, Z68.21, Z68.22, Z68.23, Z68.24 |
|  | 25.0-29.9 | 278.02, V85.21, V85.22, V85.23, V85.24, V85.25 | E66.3, Z68.25, Z68.26, Z68.27, Z68.28, Z68.29 |
|  | 30.0-34.9 | V85.30, V85.31, V85.32, V85.33, V85.34 | Z68.30, Z68.31, Z68.32, Z68.33, Z68.34 |
|  | 35.0-39.9 | V85.35, V85.36, V85.37, V85.38, V85.39 | Z68.35, Z68.36, Z68.37, Z68.38, Z68.39 |
|  | 40.0-44.9 | V85.41 | Z68.41 |
|  | 45.0-49.9 | V85.42 | Z68.42 |
|  | 50.0-59.9 | V85.43 | Z68.43 |
|  | 60.0-69.9 | V85.44 | Z68.44 |
|  | ≥70.0 | V85.45 | Z68.45 |
| 5-level BMI categorization | ≤29.9 | 783.22, 278.02, V85.0, V85.1, V85.21, V85.22, V85.23, V85.24, V85.25 | R63.6, E66.3, Z68.1, Z68.20, Z68.21, Z68.22, Z68.23, Z68.24, Z68.25, Z68.26, Z68.27, Z68.28, Z68.29 |
|  |  |  |  |
|  | 30.0-39.9 | V85.30, V85.31, V85.32, V85.33, V85.34, | Z68.30, Z68.31, Z68.32, Z68.33, Z68.34 |
|  |  | V85.35, V85.36, V85.37, V85.38, V85.39 | Z68.35, Z68.36, Z68.37, Z68.38, Z68.39 |
|  | 40.0-49.9 | V85.41, V85.42 | Z68.41, Z68.42 |
|  | 50.0-59.9 | V85.43 | Z68.43 |
|  | ≥60.0 | V85.44, V85.45 | Z68.44, Z68.45 |
| 4-level BMI categorization | ≤19.9 (underweight) | 783.22, V85.0 | R63.6, Z68.1 |
|  | 20.0-24.9 (Normal weight) | V85.1 | Z68.20, Z68.21, Z68.22, Z68.23, Z68.24 |
|  | 25.0-29.9 (Overweight) | 278.02, V85.21, V85.22, V85.23, V85.24, V85.25 | E66.3, Z68.25, Z68.26, Z68.27, Z68.28, Z68.29 |
|  | ≥30.0 (Obese) | V85.30, V85.31, V85.32, V85.33, V85.34 | Z68.30, Z68.31, Z68.32, Z68.33, Z68.34 |
|  |  | V85.35, V85.36, V85.37, V85.38, V85.39 | Z68.35, Z68.36, Z68.37, Z68.38, Z68.39 |
|  |  | V85.41, V85.42, V85.43, V85.44, V85.45 | Z68.41, Z68.42, Z68.43, Z68.44, Z68.45 |
|  |  | 278.00, 278.01, 278.03 | E66.01, E66.2, E66.09, E66.1, E66.8, E66.9 |

**eTable 3.** Determinants of having granular weight-related diagnosis codes during the 6-month preoperative period in bariatric surgery patients, 2011-2018

|  | **Odd ratio, 95% confidence interval** | |
| --- | --- | --- |
| **Determinant** | **Unadjusted^a^** | **Multivariable Adjusted^b^** |
| Type of index operation (reference SG) | | |
| AGB | 0.21 (0.18-0.23) | 0.85 (0.73-0.98) |
| RYGB | 0.69 (0.62-0.77) | 0.62 (0.55-0.70) |
| Care setting of index operation (reference Outpatient) | | |
| Inpatient | 5.18 (4.71-5.69) | 5.26 (4.62-5.99) |
| Year of index operation (reference 2011) | | |
| 2012 | 1.42 (1.23-1.64) | 1.21 (1.04-1.41) |
| 2013 | 1.92 (1.65-2.22) | 1.37 (1.16-1.61) |
| 2014 | 2.29 (1.96-2.69) | 1.61 (1.35-1.92) |
| 2015 | 3.06 (2.57-3.63) | 1.82 (1.48-2.24) |
| 2016 | 4.31 (3.57-5.21) | 1.69 (1.11-2.58) |
| 2017 | 4.93 (4.08-5.97) | 1.91 (1.25-2.93) |
| 2018 | 5.17 (3.93-6.81) | 1.79 (1.12-2.87) |
| ICD-CM coding era (reference ICD-10-CM)^c^ | | |
| ICD-9-CM | 0.34 (0.31-0.39) | 0.64 (0.44-0.92) |
| Age at index operation (reference 18-44 y) | | |
| 45-64 | 0.96 (0.87-1.06) | 0.68 (0.60-0.76) |
| 65+ | 0.52 (0.45-0.60) | 0.22 (0.18-0.27) |
| Sex (reference male) | | |
| Female | 1.41 (1.28-1.56) | 1.59 (1.41-1.78) |
| Race/ethnicity (reference White) | | |
| Asian | 0.68 (0.48-0.96) | 0.73 (0.49-1.08) |
| Black | 1.10 (0.97-1.25) | 1.02 (0.89-1.18) |
| Hispanic | 1.14 (0.98-1.32) | 1.03 (0.88-1.21) |
| Unknown | 1.48 (1.08-2.02) | 1.13 (0.81-1.57) |
| Region of residence (reference northeast) | | |
| Midwest | 0.81 (0.67-0.98) | 0.90 (0.73-1.10) |
| South | 0.53 (0.45-0.64) | 0.65 (0.54-0.79) |
| West | 0.64 (0.52-0.79) | 0.73 (0.59-0.92) |
| Type of insurance (reference Medicare Advantage) | | |
| Commercial | 0.70 (0.62-0.80) | 0.60 (0.50-0.72) |
| Charlson-Elixhauser comorbidity score (reference ≤-1) | | |
| 0 | 0.96 (0.83-1.10) | 0.82 (0.70-0.96) |
| 1+ | 0.97 (0.85-1.10) | 0.61 (0.52-0.73) |
| Number of hospital stays in the last 6 months (reference 0) | | |
| 1 | 0.42 (0.35-0.50) | 0.14 (0.07-0.26) |
| 2+ | 0.16 (0.12-0.21) | 0.09 (0.06-0.16) |
| Length of hospital stays in the last 6 months (reference 0) | | |
| 1-4 | 0.59 (0.46-0.76) | 4.48 (2.03-9.88) |
| 5+ | 0.20 (0.17-0.25) | --^d^ |
| Comorbid conditions | | |
| Diabetes | 1.76 (1.50-2.08) | 1.77 (1.45-2.15) |
| Hypertension | 1.44 (1.31-1.58) | 1.29 (1.14-1.47) |
| GERD | 1.14 (1.04-1.26) | 0.93 (0.84-1.03) |
| NAFLD | 1.71 (1.49-1.96) | 1.68 (1.44-1.96) |
| PCOS | 1.70 (1.31-2.21) | 1.30 (0.98-1.73) |
| PE | 0.74 (0.52-1.05) | 0.94 (0.62-1.40) |
| Anxiety | 1.46 (1.31-1.62) | 1.10 (0.98-1.23) |
| DVT | 0.50 (0.37-0.68) | 0.60 (0.41-0.87) |
| Depression | 1.39 (1.25-1.54) | 1.12 (1.00-1.27) |
| Dyslipidemia | 1.33 (1.21-1.45) | 1.21 (1.08-1.35) |
| Eating disorder | 1.95 (1.62-2.34) | 1.63 (1.34-1.98) |
| Infertility | --^e^ | 2.31 (0.83-6.42) |
| Kidney disease | 0.53 (0.46-0.61) | 0.50 (0.41-0.61) |
| Osteoarthritis | 1.94 (1.55-2.43) | 1.58 (1.24-2.00) |
| Psychotic disorder | 1.49 (1.17-1.89) | 1.24 (0.95-1.62) |
| Substance use disorder | 1.19 (0.94-1.50) | 0.79 (0.61-1.03) |
| Sleep apnea | 2.49 (2.26-2.75) | 2.15 (1.93-2.39) |
| Smoker | 0.66 (0.55-0.81) | 0.81 (0.65-1.00) |

Abbreviations: *AGB* adjusted gastric banding, *DVT* deep vein thrombosis, *GERD* gastroesophageal reflux disease, *NAFLD* non-alcoholic fatty liver disease, *PCOS* polycystic ovarian syndrome, *PE* pulmonary embolism, *RYGB* Roux-en-Y gastric bypass, *SG* sleeve gastrectomy

^a^ Logistic regression models included only the variables under consideration.

^b^ Logistic regression models adjusted for all variables listed in table.

^c^ The ICD-9-CM era refers to the period prior to October 1, 2015, and the ICD-10-CM era refers to the period starting from October 1, 2015.

^d^ The variable was removed from the model because it was as a linear combination of other variables included.

^e^ Estimates were not provided because cells with 10 or fewer patients have been suppressed to maintain the de-identification nature of the database.

**eTable 4.** Determinants of missing weight-related diagnosis codes in the first postoperative year in bariatric surgery patients, 2011-2018

|  | **Odd ratio, 95% confidence interval** | |
| --- | --- | --- |
| **Determinant** | **Unadjusted^a^** | **Multivariable Adjusted^b^** |
| Type of index operation (reference SG) | | |
| AGB | 0.54 (0.48-0.60) | 0.50 (0.44-0.57) |
| RYGB | 1.10 (1.04-1.17) | 0.99 (0.92-1.05) |
| Caresetting of index operation (reference Outpatient) | | |
| Inpatient | 1.34 (1.25-1.44) | 1.59 (1.45-1.74) |
| Year of index operation (reference 2011) | | |
| 2012 | 0.88 (0.79-0.98) | 0.90 (0.81-1.01) |
| 2013 | 0.95 (0.86-1.05) | 1.00 (0.90-1.11) |
| 2014 | 0.81 (0.73-0.90) | 0.80 (0.71-0.89) |
| 2015 | 0.76 (0.69-0.85) | 0.75 (0.66-0.85) |
| 2016 | 0.78 (0.70-0.86) | 0.79 (0.65-0.98) |
| 2017 | 0.64 (0.58-0.71) | 0.65 (0.53-0.81) |
| 2018 | 0.59 (0.52-0.68) | 0.61 (0.48-0.77) |
| ICD-CM coding era (reference ICD-10-CM)^c^ | | |
| ICD-9-CM | 1.29 (1.22-1.36) | 0.97 (0.82-1.16) |
| Having preoperative weight-related diagnosis (reference having specific code) | | |
| No code | 24.94 (19.36-32.12) | 23.69 (18.07-31.04) |
| Nonspecific code | 1.54 (1.37-1.74) | 1.75 (1.54-1.99) |
| Age at index operation (reference 18-44 y) | | |
| 45-64 | 0.71 (0.67-0.75) | 0.83 (0.78-0.89) |
| 65+ | 0.72 (0.65-0.80) | 0.88 (0.76-1.02) |
| Sex (reference male) | | |
| Female | 0.98 (0.92-1.05) | 0.94 (0.88-1.01) |
| Race/Ethnicity (reference White) | | |
| Asian | 1.29 (1.02-1.63) | 1.08 (0.84-1.39) |
| Black | 0.90 (0.84-0.98) | 0.84 (0.77-0.91) |
| Hispanic | 1.06 (0.97-1.15) | 1.00 (0.91-1.09) |
| Unknown | 1.01 (0.86-1.19) | 1.15 (0.97-1.37) |
| Region of residence (reference northeast) | | |
| Midwest | 1.22 (1.09-1.36) | 1.15 (1.02-1.29) |
| South | 1.78 (1.61-1.96) | 1.79 (1.61-1.98) |
| West | 1.64 (1.46-1.84) | 1.53 (1.36-1.73) |
| Type of insurance (reference Medicare Advantage) | | |
| Commercial | 1.83 (1.69-1.98) | 1.63 (1.47-1.81) |
| Charlson-Elixhauser comorbidity score (reference ≤-1) | | |
| 0 | 1.02 (0.94-1.11) | 0.95 (0.86-1.04) |
| 1+ | 0.89 (0.83-0.96) | 0.89 (0.80-0.98) |
| Number of hospital stays in the last 6 months (reference 0) | | |
| 1 | 1.31 (1.14-1.50) | 1.98 (1.14-3.43) |
| 2+ | 2.51 (1.95-3.22) | 2.31 (1.46-3.66) |
| Length of hospital stays in the last 6 months (reference 0) | | |
| 1-4 | 1.10 (0.93-1.32) | 0.55 (0.29-1.04) |
| 5+ | 2.06 (1.74-2.43) | --^d^ |
| Comorbid conditions | | |
| Diabetes | 0.57 (0.52-0.62) | 0.80 (0.72-0.89) |
| Hypertension | 0.68 (0.64-0.72) | 0.80 (0.74-0.86) |
| GERD | 1.04 (0.98-1.10) | 1.12 (1.05-1.19) |
| NAFLD | 0.88 (0.82-0.95) | 0.96 (0.88-1.04) |
| PCOS | 0.95 (0.84-1.08) | 0.81 (0.71-0.93) |
| PE | 0.74 (0.57-0.96) | 0.72 (0.54-0.97) |
| Anxiety | 0.93 (0.87-0.98) | 0.99 (0.93-1.05) |
| DVT | 0.85 (0.66-1.10) | 0.84 (0.63-1.13) |
| Depression | 0.86 (0.81-0.91) | 0.98 (0.91-1.05) |
| Dyslipidemia | 0.72 (0.68-0.76) | 0.89 (0.84-0.95) |
| Eating disorder | 0.68 (0.62-0.75) | 0.76 (0.69-0.84) |
| Infertility | 0.95 (0.66-1.35) | 0.90 (0.63-1.30) |
| Kidney disease | 0.86 (0.77-0.97) | 0.96 (0.83-1.10) |
| Osteoarthritis | 0.61 (0.55-0.69) | 0.79 (0.70-0.90) |
| Psychotic disorder | 0.76 (0.67-0.87) | 0.91 (0.79-1.04) |
| Substance use disorder | 0.93 (0.82-1.06) | 1.03 (0.89-1.20) |
| Sleep apnea | 0.64 (0.61-0.68) | 0.80 (0.75-0.85) |
| Smoker | 1.20 (1.05-1.37) | 1.05 (0.91-1.21) |

Abbreviations: *AGB* adjusted gastric banding, *DVT* deep vein thrombosis, *GERD* gastroesophageal reflux disease, *NAFLD* non-alcoholic fatty liver disease, *PCOS* polycystic ovarian syndrome, *PE* pulmonary embolism, *RYGB* Roux-en-Y gastric bypass, *SG* sleeve gastrectomy

^a^ Logistic regression models included only the variables under consideration.

^b^ Logistic regression models adjusted for all variables listed in table.

^c^ The ICD-9-CM era refers to the period prior to October 1, 2015, and the ICD-10-CM era refers to the period starting from October 1, 2015.

^d^ The variable was removed from the model because it was as a linear combination of other variables included.

**eTable 5.** Determinants of having granular weight-related diagnosis codes in the first postoperative year in bariatric surgery patients, 2011-2018

|  | **Odd ratio, 95% confidence interval** | |
| --- | --- | --- |
| **Determinant** | **Unadjusted^a^** | **Multivariable Adjusted^b^** |
| Type of index operation (reference SG) | | |
| AGB | 1.08 (1.00-1.17) | 1.97 (1.78-2.19) |
| RYGB | 0.88 (0.84-0.93) | 1.08 (1.02-1.14) |
| Caresetting of index operation (reference Outpatient) | | |
| Inpatient | 0.93 (0.88-0.99) | 0.72 (0.67-0.78) |
| Year of index operation (reference 2011) | | |
| 2012 | 1.29 (1.17-1.43) | 1.31 (1.18-1.45) |
| 2013 | 1.32 (1.20-1.45) | 1.38 (1.24-1.52) |
| 2014 | 1.69 (1.53-1.85) | 1.85 (1.67-2.05) |
| 2015 | 2.27 (2.07-2.49) | 2.37 (2.13-2.65) |
| 2016 | 2.82 (2.57-3.08) | 2.54 (2.13-3.02) |
| 2017 | 3.71 (3.40-4.06) | 3.30 (2.77-3.93) |
| 2018 | 3.94 (3.52-4.41) | 3.45 (2.86-4.16) |
| ICD-CM coding era (reference ICD-10-CM)^c^ | | |
| ICD-9-CM | 0.43 (0.41-0.45) | 0.84 (0.73-0.97) |
| Having preoperative weight-related diagnosis (reference having specific code) | | |
| No code | 0.12 (0.09-0.16) | 0.12 (0.09-0.17) |
| Nonspecific code | 0.33 (0.29-0.38) | 0.35 (0.30-0.40) |
| Age at index operation (reference 18-44 y) | | |
| 45-64 | 1.27 (1.21-1.34) | 1.08 (1.02-1.14) |
| 65+ | 1.56 (1.44-1.70) | 0.96 (0.86-1.08) |
| Sex (reference male) | | |
| Female | 1.08 (1.02-1.14) | 1.08 (1.02-1.15) |
| Race/Ethnicity (reference White) | | |
| Asian | 0.85 (0.69-1.05) | 0.92 (0.74-1.16) |
| Black | 1.08 (1.02-1.15) | 1.04 (0.98-1.12) |
| Hispanic | 1.00 (0.93-1.08) | 1.03 (0.95-1.12) |
| Unknown | 1.17 (1.02-1.34) | 0.90 (0.78-1.03) |
| Region of residence (reference northeast) | | |
| Midwest | 1.23 (1.13-1.34) | 1.27 (1.16-1.39) |
| South | 0.92 (0.85-0.99) | 0.95 (0.88-1.03) |
| West | 0.89 (0.81-0.98) | 0.94 (0.85-1.03) |
| Type of insurance (reference Medicare Advantage) | | |
| Commercial | 0.48 (0.46-0.51) | 0.58 (0.54-0.63) |
| Charlson-Elixhauser comorbidity score (reference ≤-1) | | |
| 0 | 1.06 (0.99-1.14) | 1.01 (0.94-1.10) |
| 1+ | 1.31 (1.23-1.40) | 1.01 (0.93-1.10) |
| Number of hospital stays in the last 6 months (reference 0) | | |
| 1 | 0.98 (0.87-1.11) | 0.80 (0.49-1.30) |
| 2+ | 0.90 (0.70-1.15) | 0.91 (0.61-1.37) |
| Length of hospital stays in the last 6 months (reference 0) | | |
| 1-4 | 1.03 (0.89-1.20) | 1.18 (0.67-2.05) |
| 5+ | 0.89 (0.76-1.05) | --^d^ |
| Comorbid conditions | | |
| Diabetes | 1.70 (1.59-1.81) | 1.10 (1.02-1.19) |
| Hypertension | 1.26 (1.20-1.33) | 1.12 (1.05-1.19) |
| GERD | 1.00 (0.96-1.05) | 0.93 (0.88-0.98) |
| NAFLD | 1.03 (0.97-1.09) | 1.01 (0.94-1.07) |
| PCOS | 1.08 (0.97-1.20) | 1.20 (1.07-1.35) |
| PE | 1.36 (1.12-1.66) | 1.24 (1.00-1.54) |
| Anxiety | 1.16 (1.10-1.22) | 1.02 (0.97-1.08) |
| DVT | 1.45 (1.18-1.78) | 1.35 (1.08-1.70) |
| Depression | 1.17 (1.11-1.23) | 1.05 (0.99-1.11) |
| Dyslipidemia | 1.16 (1.11-1.22) | 1.01 (0.96-1.07) |
| Eating disorder | 1.12 (1.04-1.20) | 1.08 (1.00-1.17) |
| Infertility | 1.05 (0.79-1.41) | 1.11 (0.82-1.51) |
| Kidney disease | 1.44 (1.31-1.57) | 1.21 (1.09-1.35) |
| Osteoarthritis | 1.74 (1.60-1.90) | 1.22 (1.11-1.34) |
| Psychotic disorder | 1.40 (1.27-1.55) | 1.16 (1.03-1.29) |
| Substance use disorder | 1.56 (1.40-1.74) | 1.05 (0.93-1.18) |
| Sleep apnea | 1.28 (1.22-1.34) | 1.10 (1.04-1.15) |
| Smoker | 0.67 (0.59-0.75) | 0.89 (0.78-1.01) |

Abbreviations: *AGB* adjusted gastric banding, *DVT* deep vein thrombosis, *GERD* gastroesophageal reflux disease, *NAFLD* non-alcoholic fatty liver disease, *PCOS* polycystic ovarian syndrome, *PE* pulmonary embolism, *RYGB* Roux-en-Y gastric bypass, *SG* sleeve gastrectomy

^a^ Logistic regression models included only the variables under consideration.

^b^ Logistic regression models adjusted for all variables listed in table.

^c^ The ICD-9-CM era refers to the period prior to October 1, 2015, and the ICD-10-CM era refers to the period starting from October 1, 2015.

^d^ The variable was removed from the model because it was as a linear combination of other variables included.

**eTable 6.** Performance of the modified claims-based severe obesity classification algorithm in the 6-month preoperative period (Cohort 2)

| **Classification** | **ICD-9-CM or ICD-10-CM Diagnoses** | **Sensitivity** | **Specificity** | **PPV** | **NPV** | **Kappa**  **(95% CI)** |
| --- | --- | --- | --- | --- | --- | --- |
| Having ≥1 BMI measurement ≥35.0 kg/m^2^ | V85.35, V85.36, V85.37, V85.38, V85.39, V85.41, V85.42, V85.43, V85.44, V85.45, Z68.35, Z68.36, Z68.37, Z68.38, Z68.39, Z68.41, Z68.42, Z68.43, Z68.44, Z68.45 | 100 | 71 | 100 | 78 | 0.74 (0.63, 0.85) |
| Having ≥1 BMI measurement ≥35.0 kg/m^2^ | V85.35, V85.36, V85.37, V85.38, V85.39, V85.41, V85.42, V85.43, V85.44, V85.45, Z68.35, Z68.36, Z68.37, Z68.38, Z68.39, Z68.41, Z68.42, Z68.43, Z68.44, Z68.45, 278.01, E66.01, E66.2 | 100 | 46 | 99 | 100 | 0.63 (0.49, 0.77) |
| Having ≥1 BMI measurement ≥40.0 kg/m^2^ | V85.41, V85.42, V85.43, V85.44, V85.45, Z68.41, Z68.42, Z68.43, Z68.44, Z68.45 | 98 | 75 | 96 | 83 | 0.76 (0.73, 0.79) |
| Having ≥1 BMI measurement ≥40.0 kg/m^2^ | V85.41, V85.42, V85.43, V85.44, V85.45, Z68.41, Z68.42, Z68.43, Z68.44, Z68.45, 278.01, E66.01, E66.2 | 100 | 5 | 87 | 100 | 0.09 (0.05, 0.12) |

Abbreviations: *BMI* body mass index, *ICD* international classification of diseases, *PPV* positive predictive value, *NPV* negative predictive value, *CI* confidence interval

**eTable 7.** Performance of the modified claims-based body mass index (BMI) categorization algorithm in the 6-month preoperative (Cohort 2) and 1-year postoperative periods (Cohort 3)

| **BMI categories, kg/m^2^** | **ICD-9-CM or ICD-10-CM Diagnoses** | **Last preoperative BMI^a^** | | | | | **Last postoperative BMI^b^** | | | | |
| --- | --- | --- | --- | --- | --- | --- | --- | --- | --- | --- | --- |
|  |  | **Sensitivity** | **Specificity** | **PPV** | **NPV** | **Kappa** | **Sensitivity** | **Specificity** | **PPV** | **NPV** | **Kappa** |
| ≤19.9 | 783.22, V85.0, R63.6, Z68.1 | 100 | 100 | 80 | 100 | 0.78  (0.76, 0.79) | 100 | 100 | 100 | 100 | 0.84 (0.80,0.87) |
| 20.0-24.9 | V85.1, Z68.20-Z68.24 | 60 | 100 | 100 | 100 |  | 75 | 100 | 100 | 100 |  |
| 25.0-29.9 | 278.02, V85.21-V85.25, E66.3, Z68.25-Z68.29 | 70 | 100 | 100 | 100 |  | >82^c^ | >98^c^ | >82^c^ | 98 |  |
| 30.0-34.9 | V85.30-V85.34, Z68.30-Z68.34 | 30 | 100 | >62^c^ | 99 |  | 84 | >97^c^ | >93^c^ | 93 |  |
| 35.0-39.9 | V85.35-V85.39, Z68.35-Z68.39 | 64 | 97 | 81 | 93 |  | 84 | 94 | 81 | 95 |  |
| 40.0-44.9 | V85.41, Z68.41 | 76 | 87 | 71 | 90 |  | 76 | 93 | 68 | 95 |  |
| 45.0-49.9 | V85.42, Z68.42 | 71 | 90 | 65 | 92 |  | 72 | 97 | 73 | 97 |  |
| 50.0-59.9 | V85.43, Z68.43 | 79 | 93 | 76 | 94 |  | >65^c^ | 97 | 61 | >98^c^ |  |
| 60.0-69.9 | V85.44, Z68.44 | 70 | 98 | 67 | 98 |  | 100 | 99 | 17 | 100 |  |
| ≥70.0 | V85.45, Z68.45 | 53 | 100 | 63 | 99 |  | --^d^ | 100 | 0 | 100 |  |
|  |  |  |  |  |  |  |  |  |  |  |  |
| ≤29.9 | 783.22, 278.02, V85.0, V85.1, V85.21-V85.25, R63.6, Z68.1, E66.3, Z68.20-Z68.29 | 87 | 100 | 100 | 100 | 0.77  (0.76, 0.79) | >84^c^ | >98^c^ | >84^c^ | 98 | 0.82  (0.78, 0.87) |
| 30.0-39.9 | V85.30-V85.39, Z68.30-Z68.39 | 67 | 98 | 90 | 93 |  | 89 | 93 | 94 | 87 |  |
| 40.0-49.9 | V85.41, V85.42, Z68.41, Z68.42 | 89 | 81 | 82 | 88 |  | 86 | 93 | 80 | 95 |  |
| 50.0-59.9 | V85.43, Z68.43 | 79 | 93 | 76 | 94 |  | >65^c^ | 97 | 61 | >98^c^ |  |
| ≥60.0 | V85.44, V85.45, Z68.44, Z68.45 | 75 | 98 | 74 | 98 |  | 100 | 99 | 14 | 100 |  |
|  |  |  |  |  |  |  |  |  |  |  |  |
| ≤19.9 | 783.22, V85.0, R63.6, Z68.1 | 100 | 100 | 80 | 100 | 0.90  (0.83, 0.97) | 100 | 100 | 100 | 100 | 0.90  (0.84, 0.96) |
| 20.0-24.9 | V85.1, Z68.20-Z68.24 | 50 | 100 | 100 | 100 |  | 75 | 100 | 100 | 100 |  |
| 25.0-29.9 | 278.02, V85.21-V85.25, E66.3, Z68.25-Z68.29 | 58 | 100 | 100 | 100 |  | >82^c^ | >98^c^ | >82^c^ | 98 |  |
| ≥30.0 | 278.00, 278.01, 278.03, V85.30-V85.39, V85.41-V85.45, E66.01, E66.09, E66.1, E66.2, E66.8, E66.9, Z68.30-Z68.39, Z68.41-Z68.45 | 100 | 77 | 100 | 100 |  | >98^c^ | >81^c^ | 98 | >81^c^ |  |

Abbreviations: *BMI* body mass index, *ICD* international classification of diseases, *PPV* positive predictive value, *NPV* negative predictive value, *CI* confidence interval

^a^ The last available weight-related diagnosis code in claims in the 6-month preoperative period was compared with the last available BMI measurement in the electronic health records (EHR) during the same period among Cohort 2 patients.

^b^ The last available weight-related diagnosis code in claims in the 1-year postoperative period was compared with the last available BMI measurement in the EHR during the same period among Cohort 3 patients.

^c^ Cells with 10 or fewer patients are suppressed to maintain the de-identification nature of the database.

^d^ Sensitivity was not calculated because no patients had relevant BMI measurement at this level in the EHR.

**eFigure 4.** The sample size and estimated weighted kappa when relaxing the proximity restriction between the weight-related diagnosis codes and the body mass index (BMI) measurement in the electronic health records for categorization of the last available BMI in the first postoperative year using the 10-level BMI categorization algorithm. AGB = adjusted gastric banding; RYGB = Roux-en-Y gastric bypass; SG = sleeve gastrectomy.

**eTable 8.** Performance of the claims-based body mass index (BMI) categorization algorithm for the last available BMI in different postoperative periods

| **Length of follow-up period (month)** | **Sample size** | **Weighted kappa, 95% confidence interval** | | |
| --- | --- | --- | --- | --- |
|  |  | **10-level** | **5-level** | **4-level** |
| 6 | 581 | 0.82 (0.79, 0.85) | 0.81 (0.78, 0.85) | 0.82 (0.78, 0.85) |
| 12 | 511 | 0.84 (0.80,0.87) | 0.82 (0.78, 0.87) | 0.90 (0.84, 0.96) |
| 24 | 224 | 0.86 (0.80, 0.92) | 0.86 (0.80, 0.92) | 0.85 (0.80, 0.92) |
